# Quality of Life of Vietnamese Ostomy Patients: A Multidimensional Assessment Using the City of Hope Quality of Life-Ostomy Questionnaire (CoH-QoL-OQ)

**DOI:** 10.7759/cureus.109936

**Published:** 2026-05-30

**Authors:** Loc H Tran, Hai V Nguyen, Anh T Nguyen, Phuong H Chung, Duy Q Ngo

**Affiliations:** 1 Digestive Surgery Department, Nhan dan Gia Dinh Hospital, Ho Chi Minh City, VNM; 2 General Surgery Department, School of Medicine, University of Medicine and Pharmacy at Ho Chi Minh City, Ho Chi Minh City, VNM

**Keywords:** city of hope quality of life-ostomy questionnaire (coh-qol-oq), colostomy, ileostomy, patient-reported outcome measures, quality of life

## Abstract

Background: Ostomy creation is a life-saving intervention for gastrointestinal and abdominopelvic conditions but is associated with substantial changes in quality of life. This study evaluated the quality of life among ostomy patients using a validated multidimensional instrument.

Methods: A cross-sectional study was conducted at a tertiary hospital in Vietnam. Clinical data included demographic characteristics, underlying diseases, and stoma-related features. A subset of patients completed the City of Hope Quality of Life-Ostomy Questionnaire (CoH-QoL-OQ). Additional data on daily life, including occupational status, marital status, caregiving, and mobility, were collected through structured interviews. Quality of life was assessed across four domains, including physical, psychological, social, and spiritual well-being.

Results: Among 552 patients, 87 patients in the interview cohort had comparable baseline and stoma-related characteristics to those of the overall cohort, suggesting that the interview cohort was broadly representative of the overall population. Overall quality of life was favorable, with a mean score of 8.26. Spiritual well-being was highest at 9.16, whereas social well-being was lowest at 7.02. Many patients maintained work, travel, and social activities, while others experienced limitations in occupational participation and mobility. Marital relationships remained stable. Stoma duration was the most relevant factor associated with improved outcomes.

Conclusion: Patients with a stoma can achieve a relatively favorable quality of life through gradual adaptation. Daily life outcomes, including work and social participation, are key components of well-being, highlighting the need for long-term, patient-centered care.

## Introduction

For a wide range of gastrointestinal and abdominopelvic conditions, ostomy creation remains a fundamental surgical intervention that preserves essential physiological function while contributing to improved disease control and survival [1]. Depending on the underlying indication, a stoma may be constructed as either temporary or permanent; however, irrespective of its intended duration, it inevitably imposes substantial and multidimensional changes in patients’ daily functioning, psychological well-being, social interactions, and body image perception [2]. As a result, successful adaptation extends beyond technical surgical outcomes and requires sustained medical, nursing, and psychosocial support within a coordinated multidisciplinary framework aimed at optimizing overall quality of life.

In parallel with advances in surgical care, quality of life has emerged as a critical endpoint complementing traditional clinical indicators. Contemporary evaluation of treatment effectiveness increasingly incorporates patient-reported outcomes, reflecting a shift toward holistic, patient-centered care. Within this context, improvements in quality of life not only indicate therapeutic success but also reflect the adequacy of long-term support systems and the patient’s capacity for functional and psychosocial adaptation. To facilitate such assessment in patients with a stoma, the City of Hope Quality of Life-Ostomy Questionnaire (CoH-QoL-OQ), developed in 1983 in the United States, provides a multidimensional framework encompassing physical, psychological, social, and spiritual domains, thereby enabling a comprehensive evaluation of post-stoma adaptation [2].

The CoH-QoL-OQ has been widely applied across diverse cultural settings and translated into multiple languages, including Chinese [3], Persian (Iran) [4], Malayalam (India) [5], Croatian [6], Amharic (Ethiopia) [7], Turkish [8], Portuguese (Brazil) [9], Arabic [10], and other languages. Validation studies in these populations have consistently demonstrated their feasibility, reliability, and construct validity, while also revealing considerable variability in both domain-specific and overall quality of life outcomes [3-6]. Such differences likely reflect the influence of cultural norms, healthcare accessibility, and the extent of family and community support, underscoring the context-dependent nature of quality of life as a multidimensional construct.

In Vietnam, ostomy formation is increasingly encountered in routine gastrointestinal surgical practice, particularly in the management of colorectal malignancies and other abdominopelvic conditions. Despite this growing clinical relevance, evidence regarding the quality of life of Vietnamese ostomy patients remains limited, and few data are available to guide long-term rehabilitation and psychosocial support strategies. Therefore, the present study, entitled “Quality of Life of Vietnamese Ostomy Patients: A Multidimensional Assessment Using the CoH-QoL-OQ”, was conducted to assess multidimensional quality of life among Vietnamese ostomy patients using the CoH-QoL-OQ questionnaire, with a focus on physical, psychological, social, and spiritual well-being. In addition, the study explored selected clinical and demographic factors associated with quality of life outcomes, including the relationship between stoma duration and domain-specific quality of life scores. The findings of this study may provide preliminary data regarding the multidimensional quality of life of patients living with an ostomy.

## Materials and methods

Study design and setting

This study was designed as a cross-sectional study conducted at a tertiary referral hospital in Vietnam. The study aimed to evaluate the quality of life of patients with a stoma and to identify factors associated with quality of life outcomes using a standardized instrument.

Clinical data were retrospectively collected from hospital medical records, including both paper-based records and electronic medical systems. In parallel, a subset of patients was recruited using a convenience sampling method for direct interviews to assess quality of life using a validated questionnaire. The study combined retrospective clinical data with prospective patient-reported outcomes to provide a comprehensive evaluation. Additional data on daily life and social functioning were also collected through structured interviews.

Study population

The study population included all patients who underwent stoma creation for gastrointestinal or abdominopelvic conditions during the study period and met the inclusion criteria. A subset of patients was recruited into the interview cohort using a convenience sampling method and completed the CoH-QoL-OQ questionnaire. Data collection and direct patient interviews were conducted from July 2025 to February 2026. Most patients in the interview cohort had undergone ostomy creation between Jan 2023 and Dec 2025, whereas a small number of patients who had previously undergone definitive procedures, such as abdominoperineal resection, had lived with a permanent stoma for more than 10 years and were encountered sporadically during the study period.

Patients in the interview cohort were primarily recruited when returning to the hospital for various reasons, most commonly for stoma closure, postoperative surveillance following colorectal cancer surgery, or other follow-up consultations. Interviews were conducted exclusively by the principal investigator through direct face-to-face one-on-one interaction. Nearly all eligible patients agreed to participate in the study, with only two patients declining the interview. No enrolled participant was excluded because of incomplete or missing essential data.

Inclusion criteria comprised patients who underwent stoma creation, including either colostomy or ileostomy. For the interview cohort, eligible participants were those who had been discharged after stoma formation, had lived at home for a certain period, were able to reliably respond to interview questions, and provided informed consent to participate in the study. Exclusion criteria included patients with incomplete or missing key clinical data.

Data collection

Data were collected from two main sources: medical records and direct patient interviews.

Clinical variables extracted from medical records included demographic characteristics (age and sex), American Society of Anesthesiologists (ASA) classification, cancer status, underlying diagnosis, surgical approach, urgency of stoma creation, bowel segment used for stoma, stoma type (anatomical and duration), and stoma configuration.

For the interview cohort, additional data related to patient experience were collected through structured interviews. These included occupational changes, marital status, caregiving arrangements, mobility and travel activity, duration of stoma, stoma location, type of pouching system, and stoma-related complications.

Quality of life assessment using the CoH-QoL-OQ

Quality of life was assessed using the CoH-QoL-OQ, a validated instrument specifically designed for patients with a stoma. The questionnaire consists of 43 items divided into four domains: physical well-being (items 1-11), psychological well-being (items 12-24), social well-being (items 25-36), and spiritual well-being (items 37-43). To ensure consistent interpretation of the questionnaire, negatively worded items were reverse-coded prior to analysis according to the original CoH-QoL-OQ scoring guidelines, such that higher scores consistently reflected better quality of life. Reverse-coded items included items 1-12, 15, 18-19, 22-30, 32-34, and 37. Domain scores were calculated as the mean of items within each domain, whereas the overall quality of life score was calculated as the mean of all items. Descriptive statistics, including mean, standard deviation, median, and range, were used to summarize quality of life scores [11].

The CoH-QoL-OQ is a validated instrument with established reliability and validity in previous studies [3-6]. In this study, the questionnaire was translated using a forward-backward translation method to ensure linguistic and conceptual equivalence. The translation process involved consultation with a native English speaker to enhance language accuracy and cultural appropriateness. A pilot test was subsequently conducted on 10 patients to evaluate the clarity, comprehensibility, and applicability of the translated version, and minor adjustments were made accordingly (Appendix A).

Statistical analysis

Statistical analysis was performed using IBM SPSS Statistics (version 20.0; IBM Corp., Armonk, NY, USA). Continuous variables were summarized using mean, standard deviation, median, and range, while categorical variables were presented as frequencies and percentages. Comparisons between the interview cohort and the non-interview cohort were performed using appropriate statistical tests. Categorical variables were analyzed using the chi-square test, whereas continuous variables were compared using an independent samples t-test or analysis of variance (ANOVA) as appropriate. A p-value < 0.05 was considered statistically significant.

Ethical considerations

The study was conducted in accordance with the principles of the Declaration of Helsinki. This study was approved by the Institutional Review Board of Nhan dan Gia Dinh Hospital, Ho Chi Minh City, Vietnam (approval No. 94/NDGĐ-HĐĐĐ, dated June 16, 2025).

Patient confidentiality was strictly maintained, and all data were anonymized during analysis. For the interview cohort, written informed consent was obtained from all participants before participation.

## Results

A total of 552 patients who underwent stoma creation were included in the study. Among them, 87 patients were recruited into the interview cohort using a convenience sampling method and completed the CoH-QoL-OQ questionnaire.

Table 1 demonstrates that the interview cohort was comparable to the overall cohort across most baseline and stoma-related characteristics, suggesting a broadly representative sample. The age distribution was similar between groups, with most patients in the 40-70-year range, followed by older individuals, while younger patients accounted for a smaller proportion. Key variables, including ASA classification, cancer status, underlying diagnosis, surgical approach, urgency of stoma creation, bowel segment used, and stoma type, both anatomical and duration, were similarly distributed between the two groups. In both cohorts, colorectal cancer was the most common indication for stoma formation, and temporary stomas were more common than permanent ones. Emergency procedures accounted for the majority of cases.

**Table 1:** Comparison of baseline characteristics and stoma-related features between patients in the interview cohort (who completed the COH-QoL-OQ) and those in the non-interview cohort ASA: American Society of Anesthesiologists, CoH-QoL-OQ: City of Hope Quality of Life-Ostomy Questionnaire ^c^ The chi-square test (χ² test) was applied to evaluate differences between groups.

Characteristics	Interview cohort	Non-interview cohort	Overall cohort	Test statistic values	p-value
	(N = 87)	(N = 465)	(N = 552)		
	n (%)	n (%)	n (%)		
Age group (years)				χ² = 4.60	0.10^c^
< 40 years	5 (5.7)	26 (5.6)	31 (5.6)		
40–70 years	62 (71.3)	278 (59.8)	340 (61.6)		
> 70 years	20 (23.0)	161 (34.6)	181 (32.8)		
Sex				χ² = 4.48	0.03^c^
Male	58 (66.7)	253 (54.4)	311 (56.3)		
Female	29 (33.3)	212 (45.6)	241 (43.7)		
ASA classification				χ² = 1.87	0.39^c^
I	5 (5.7)	14 (3)	19 (3.4)		
II	9 (10.3)	59 (12.7)	68 (12.3)		
III	73 (83.9)	392 (84.3)	465 (84.2)		
Cancer status				χ² = 1.04	0.31^c^
No	30 (34.5)	135 (29)	165 (29.9)		
Yes	57 (65.5)	330 (71)	387 (70.1)		
Diagnosis group					
Colorectal cancer	55 (63.2)	316 (68)	371 (67.2)		
Other cancers	2 (2.3)	14 (3)	16 (2.9)		
Benign bowel disease	24 (27.6)	106 (22.8)	130 (23.6)		
Anorectal disease	3 (3.4)	13 (2.8)	16 (2.9)		
Others	3 (3.4)	16 (3.4)	19 (3.4)		
Surgical approach				χ² = 3.68	0.16^c^
Laparoscopic surgery	44 (50.6)	184 (39.6)	228 (41.3)		
Open surgery	40 (46.0)	263 (56.6)	303 (54.9)		
Local procedure	3 (3.4)	18 (3.9)	21 (3.8)		
Urgency of stoma creation				χ² = 3.10	0.08^c^
Elective	23 (26.4)	85 (18.3)	108 (19.6)		
Emergency	64 (73.6)	380 (81.7)	444 (80.4)		
Bowel segment used for the stoma					
Sigmoid colon	42 (48.3)	164 (35.3)	206 (37.3)		
Descending colon	15 (17.2)	83 (17.8)	98 (17.8)		
Transverse colon	5 (5.7)	81 (17.4)	86 (15.6)		
Ascending colon	1 (1.1)	0 (0)	1 (0.2)		
Cecum	2 (2.3)	8 (1.7)	10 (1.8)		
Ileum	22 (25.3)	129 (27.7)	151 (27.4)		
Type of stoma (anatomical)				χ² = 4.48	0.64^c^
Colostomy	65 (74.7)	336 (72.3)	401 (72.6)		
Ileostomy	22 (25.3)	129 (27.7)	151 (27.4)		
Type of stoma (duration)				χ² = 1.13	0.29^c^
Temporary	74 (85.1)	414 (89)	488 (88.4)		
Permanent	13 (14.9)	51 (11)	64 (11.6)		
Stoma configuration				χ² = 15.51	< 0.01^c^
End	49 (56.3)	168 (36.1)	217 (39.3)		
Loop	33 (37.9)	282 (60.6)	315 (57.1)		
Blowhole	5 (5.7)	15 (3.2)	20 (3.6)		

Some differences were observed between the two groups, mainly in sex distribution and stoma configuration. The interview cohort had a higher proportion of male patients compared with the overall cohort. Regarding stoma configuration, end stomas were more frequently observed in the interview cohort, whereas loop stomas were more common in the non-interview cohort. These variations were limited to specific variables, while other baseline and stoma-related characteristics remained comparable between groups. No notable differences were identified in the remaining variables. Generally, the interview cohort reflects the broader study population to a reasonable extent.

Table 2 provides a structured overview of the lived experiences of patients in the interview cohort, reflecting the multidimensional changes following stoma creation (Appendix B). Regarding occupational status, approximately half of the patients were retired or not employed before surgery, while the remaining participants were nearly evenly divided between those who maintained their previous work and those who experienced changes after stoma formation. Marital status remained stable during the study period, with most patients being married and no reported changes in either the married or unmarried groups. In terms of caregiving, most patients performed self-care or shared care with family members, while reliance on healthcare staff was minimal, highlighting the central role of patient autonomy alongside family support in long-term stoma management.

**Table 2:** Patient experience and care-related factors among interviewed ostomy patients

Characteristics	Interview cohort (N =87)
	n (%)
Change in occupational status	
No	22 (25.3)
Yes	21 (24.1)
Retired or not employed before surgery	44 (50.6)
Marital status	
Married	58 (66.7)
Other (single, divorced, or widowed)	29 (33.3)
Change in marital status (married participants)	
None	58/58 (100)
Yes	0 (0)
Change in marital status (unmarried participants)	
None	29/29 (100)
Yes	0 (0)
Primary caregiver	
Self	35 (40.2)
Spouse	16 (18.4)
Other family member(s)	27 (31)
Self and family	7 (8)
Healthcare staff	2 (2.3)
Mobility and travel activity	
Regular travel	16 (18.4)
Homebound	37 (42.5)
Short local trips	26 (29.9)
Rarely traveled before surgery	8 (9.2)
Duration of stoma	
< 1 month	13 (14.9)
1–3 months	17 (19.5)
3–12 months	41 (47.1)
> 12 months	16 (18.4)
Stoma location	
Left lower quadrant	59 (67.8)
Right lower quadrant	22 (25.3)
Left upper quadrant	6 (6.9)
Type of pouching system	
One-piece system	52 (59.8)
Two-piece system	32 (36.8)
Belt and rubber glove	1 (1.1)
Belt and plastic bag	2 (2.3)
Stoma-related complications	
None	65 (74.7)
Parastomal hernia	10 (11.5)
Peristomal skin ulceration	5 (5.7)
Bleeding	4 (4.6)
Prolapse	2 (2.3)
Retraction	1 (1.1)

Daily functioning and stoma-related characteristics demonstrate varying levels of adaptation among patients. Some individuals maintained regular mobility and travel, whereas a considerable proportion were limited to home or short-distance activities, suggesting different degrees of restriction in social participation. The duration of stoma varied across patients, with the largest group having lived with a stoma for several months, alongside smaller groups at earlier and longer stages. Most stomas were located in the left lower quadrant, and one-piece pouching systems were more commonly used than two-piece systems. In addition, a small number of patients used improvised solutions such as plastic bags or rubber gloves. The majority of patients did not report stoma-related complications; among those who did, parastomal hernia and peristomal skin complications were the most common, while other complications were relatively uncommon. Overall, these findings reflect a spectrum of adaptation, ranging from preserved independence to varying degrees of limitation in daily life.

Table 3 summarizes the quality of life scores across the four domains of the CoH-QoL-OQ, together with the overall score. Overall, the mean quality of life was relatively high, indicating a generally favorable level of adaptation among patients living with a stoma. Among the four domains, spiritual well-being recorded the highest mean score with the least variability, suggesting a consistently strong sense of spiritual or existential support across the cohort. Physical well-being also demonstrated high scores, reflecting good functional status and effective symptom control in most patients. Although psychological well-being was slightly lower, it remained at a favorable level, indicating that emotional well-being remained relatively favorable in many patients (Appendix C).

**Table 3:** Mean scores of quality of life domains assessed by the COH-QoL-OQ CoH-QoL-OQ: City of Hope Quality of Life-Ostomy Questionnaire

Domain	Number of items	Mean	Standard deviation	95% confidence interval	Median		Min	Max
Physical well-being	11	8.76	1.18	(8.51 – 9.01)		9.09	3.18	10
Psychological well-being	13	8.10	1.66	(7.75 – 8.46)		8.38	2.92	10
Social well-being	12	7.02	1.91	(6.61 – 7.42)		7.20	2.82	10
Spiritual well-being	7	9.16	0.82	(8.99 – 9.34)		9.40	6.50	10
Overall well-being	43	8.26	1.13	(8.02 – 8.50)		8.46	4.36	9.96

In contrast, social well-being emerged as the most affected domain, characterized by the lowest mean score and the greatest variability. This pattern suggests considerable heterogeneity in social functioning, with some patients maintaining satisfactory social engagement while others experience notable difficulties. Median values were generally close to their corresponding means across all domains, indicating a relatively symmetrical distribution of scores. However, the wide range observed, particularly in the social and psychological domains, highlights the presence of a subgroup of patients facing ongoing challenges. Taken together, these findings suggest that overall quality of life was well preserved, with particularly strong adaptation in the spiritual and physical domains, whereas social well-being remained the most vulnerable aspect.

Table 4 presents the associations between patient characteristics and quality of life scores across all domains and overall. In general, most baseline variables, including age group, sex, cancer status, and stoma type (both anatomical and duration), were not significantly associated with quality of life, suggesting a relatively consistent level of well-being across different patient subgroups.

**Table 4:** Association between patient characteristics and quality of life scores ^a^ One-way analysis of variance (ANOVA) was used to evaluate differences between groups. ^t^ The independent-samples t-test was used to evaluate differences between groups. A p-value < 0.05 was considered statistically significant.

Characteristics	Number of patients (n)	Physical	Psychological	Social	Spiritual	Overall
Age group (years)						
< 40 years	5	8.82	8.57	7.53	8.93	8.47
40–70 years	62	8.62	8.08	7	9.11	8.21
> 70 years	20	9.18	8.03	6.93	9.39	8.38
Test statistic values and p-value		F = 1.71; p = 0.19^a^	F = 0.26; p = 0.81^a^	F = 0.20; p = 0.82^a^	F = 1.06; p = 0.35^a^	F = 0.27; p = 0.76^a^
Sex						
Male	58	8.83	7.9	6.83	9.12	8.17
Female	29	8.61	8.51	7.39	9.25	8.44
Test statistic values and p-value		t = 0.81; p = 0.42^t^	t = -1.63; p = 0.11^t^	t = -1.30; p = 0.20^t^	t = -0.73; p = 0.47^ t^	t = -1.06; p = 0.29^ t^
Cancer status						
No	30	8.59	7.89	6.99	9.09	8.14
Yes	57	8.85	8.21	7.03	9.2	8.33
Test statistic values and p-value		t = -1.00; p = 0.32^t^	t = -0.87; p = 0.39^ t^	t = -0.10; p = 0.91^t^	t = -0.62; p = 0.54^ t^	t = -0.73; p = 0.47^ t^
Type of stoma (anatomical)						
Colostomy	65	8.8	8.13	7.13	9.2	8.32
Ileostomy	22	8.64	8.02	6.67	9.07	8.1
Test statistic values and p-value		t = 0.57; p = 0.57^t^	t = 0.27; p = 0.79^ t^	t = 1.00; p = 0.32^t^	t = 0.63; p = 0.53^ t^	t = 0.78; p = 0.44^ t^
Type of stoma (duration)						
Temporary	74	8.79	8.01	6.96	9.16	8.23
Permanent	13	8.6	8.6	7.33	9.21	8.44
Test statistic values and p-value		t = 0.52; p = 0.60^t^	t = -1.17; p = 0.24^ t^	t = -0.65; p = 0.52^t^	t = -0.21; p = 0.84^ t^	t = -0.60; p = 0.55^ t^
Duration of stoma						
< 1 month	13	7.99	7.6	6.04	8.58	7.55
1–3 months	17	8.53	7.6	6.22	9.22	7.89
3–12 months	41	8.99	8.25	7.37	9.3	8.48
> 12 months	16	9.04	8.66	7.75	9.24	8.67
Test statistic values and p-value		F = 3.14; p = 0.03^a^	F = 1.67; p = 0.18^a^	F = 3.70; p = 0.02^a^	F = 2.80; p = 0.04^a^	F = 3.86; p = 0.01^a^

In contrast, the duration of stoma showed a significant association with several domains of quality of life. Patients with longer stoma duration tended to report higher scores in physical, social, and spiritual well-being, as well as in overall quality of life, suggesting a possible association between longer stoma duration and more favorable QoL scores. Although psychological well-being also demonstrated an increasing trend, this association did not reach statistical significance. Across domains, social well-being consistently recorded the lowest scores, whereas spiritual well-being remained the highest, mirroring the pattern observed in Table 3. Taken together, these findings suggest that while most demographic and clinical factors had limited impact on quality of life, stoma duration appears to be the most relevant factor associated with improved adaptation and overall well-being.

## Discussion

A total of 552 patients who underwent stoma creation were included in this study, providing a broad overview of the clinical spectrum encountered in a tertiary referral setting. The majority of patients were in the middle-aged to elderly groups, which is consistent with the epidemiological profile of conditions requiring stoma formation, particularly colorectal malignancies. In addition, most stomas were created in emergency settings, reflecting the acute and often severe nature of the underlying diseases. These findings are in line with previous studies, including those by Mayadevi et al. (2019) in India [5] and Vural et al. (2021) in Türkiye [8], both of which reported that colorectal cancer and acute abdominal conditions remain the leading indications for stoma creation, with a predominance of emergency procedures. From a clinical standpoint, these results further emphasize the role of stoma formation as a life-saving intervention and highlight the importance of optimizing perioperative care in high-risk surgical populations.

The interview cohort was comparable to the overall study population across most baseline and stoma-related characteristics. This finding indicates that the interviewed sample was broadly representative, despite the use of a convenience sampling approach. The consistency observed between the two groups suggests that major selection bias is unlikely and supports the internal validity of the study. A possible explanation is that patients included in the interview cohort were primarily those returning for follow-up visits or stoma closure, which reflects routine clinical pathways. Similar recruitment strategies have been reported in previous studies using the CoH-QoL-OQ, particularly in the work of Grant et al. (2004) in the United States [2], where follow-up cohorts were utilized to assess long-term quality of life. In real-world practice, this level of representativeness enhances the applicability of the findings to everyday surgical practice.

However, several differences were identified between the two groups, particularly in sex distribution and stoma configuration. As these differences reached statistical significance, they may have implications for the interpretation of the results, especially given that quality of life can vary according to patient characteristics and clinical context. Notably, the higher proportion of male patients in the interview cohort remains without a clear explanation and may reflect the influence of multiple interacting factors. Patients with end stomas, often created as part of definitive procedures such as Hartmann’s operation or abdominoperineal resection, are more likely to return for planned follow-up, including consideration of stoma reversal or long-term oncological surveillance. In addition, loop stomas are frequently constructed as temporary or palliative measures, particularly in emergency settings or advanced malignancy, and these patients are typically less likely to return for follow-up once the acute condition has resolved or when care is shifted toward palliation. These differences between end and loop stomas, together with variations in treatment goals between curative and palliative approaches, may contribute to heterogeneous follow-up patterns across subgroups. From a practical standpoint, this suggests that certain patient populations, particularly those with temporary or palliative stomas, may be underrepresented, highlighting the need for more comprehensive and structured follow-up strategies in future studies to minimize potential selection bias.

Occupational outcomes following stoma creation showed a variable pattern among patients. While approximately half of the cohort had already been retired or not employed prior to surgery, the remaining patients were nearly evenly divided between those who maintained their occupational status and those who experienced changes after stoma formation. This finding suggests that the impact of a stoma on occupational functioning is not uniform but varies across individuals. One possible explanation is that the ability to continue working depends on multiple factors, including physical condition, type of occupation, and confidence in stoma management. Similar variability has been reported in previous studies, where some patients successfully returned to work while others faced difficulties due to physical limitations or social concerns. For example, the study by Mayadevi et al. (2019) [5] reported that 10 out of 24 patients (41.7%) experienced changes in occupation related to living with a stoma, supporting the heterogeneous impact of stoma formation on work-related outcomes. From an applied perspective, these findings highlight the importance of individualized support and vocational counseling to facilitate reintegration into the workforce after surgery.

Notably, marital status remained completely stable throughout the study period, with no reported changes among either married or unmarried participants. This finding is noteworthy, as it suggests that intimate relationships are largely preserved despite the physical and psychological challenges associated with living with a stoma. The absence of change may reflect strong family support systems. In addition, the relatively high proportion of married patients, accounting for approximately two-thirds of the cohort, is consistent with findings reported by Konjevoda et al. (2020) in Croatia [6]. One possible explanation is that patients and their partners gradually adapt together to the new condition, maintaining emotional and social bonds over time. Although limited studies have specifically reported marital stability in ostomy populations, existing literature consistently emphasizes the critical role of family support in improving quality of life. In the context of patient care, this finding underscores the importance of involving family members in patient education and counseling, as they represent a key resource in the long-term adaptation process.

A notable finding of this study is the heterogeneity in patients’ ability to maintain daily and social activities following stoma creation. While some individuals were able to continue traveling, engaging in social interactions, and participating in religious practices such as attending church services or visiting pagodas, others experienced varying degrees of limitation or discontinuation of these activities. This variation indicates that adaptation to life with a stoma is not uniform but differs substantially across individuals. A possible explanation is that functional capacity, psychological readiness, and confidence in stoma management may influence the extent to which patients resume normal activities. In routine clinical settings, this finding highlights the need for individualized rehabilitation strategies, including both physical and psychosocial support, to facilitate reintegration into daily life.

Overall quality of life in this cohort was relatively favorable, indicating that many patients were able to adapt effectively after stoma creation. This finding suggests that, despite the significant physical and psychosocial changes associated with a stoma, patients can achieve a satisfactory level of well-being over time. One possible explanation is that gradual adaptation, combined with improved self-care skills and support from family members, contributes to this positive outcome. This finding is consistent with previous international studies using the CoH-QoL-OQ instrument, including those by Grant et al. (2004) in the United States [2], Anaraki et al. (2014) in Iran [4], Zewude et al. (2021) in Ethiopia [7], Konjevoda et al. (2020) in Croatia [6], Costa et al. (2023) in Brazil [9], and Reem Awad Alharbi et al. (2023) in Saudi Arabia [10], all of which reported generally favorable quality of life among patients living with a stoma, albeit with some variation across populations and healthcare settings. In clinical practice, these results reinforce the importance of long-term follow-up, patient education, and supportive care to sustain and further improve quality of life.

Across the four domains, spiritual well-being consistently demonstrated the highest scores, suggesting that many patients were able to achieve a sense of adaptation and inner stability over time. This favorable pattern may be partly explained by community and familial support systems, particularly in the Vietnamese context, where values such as acceptance, inner peace, and letting go in the face of illness are strongly emphasized. Supporting patients in maintaining spiritual practices and integrating spiritual care into comprehensive management, as suggested by Diniz et al. (2021) in Brazil [12], may further enhance overall well-being. In contrast, social well-being remained the most affected domain, indicating that difficulties in social interaction and reintegration persist despite psychological and spiritual adaptation. Factors such as altered body image, concerns about leakage, and perceived stigma may hinder full participation in social activities. This pattern is consistent with previous studies, including those by Anaraki et al. (2014) in Iran [4], Zewude et al. (2021) in Ethiopia [7], and Konjevoda et al. (2020) in Croatia [6], all of which reported relatively lower scores in the social domain compared with other domains. Clinically, these findings highlight the importance of targeted interventions to improve social reintegration, alongside a holistic approach that includes spiritual support.

The duration of the stoma emerged as the most relevant factor associated with quality of life, with longer duration linked to improved outcomes across multiple domains. This finding suggests a progressive process of adaptation, in which patients gradually develop self-care skills, confidence, and psychological resilience. A plausible explanation is that repeated exposure to daily stoma management reduces anxiety and enhances coping capacity over time. Similar trends have been described in previous studies, where quality of life improves as patients become more accustomed to living with a stoma. Interestingly, although psychological well-being showed a positive trend, the association did not reach statistical significance, indicating that emotional adaptation may be influenced by additional factors such as personality traits and social support. From a management perspective, these results emphasize the importance of early postoperative education and continuous support, particularly during the initial adaptation period.

This study has several limitations that should be acknowledged. First, the use of convenience sampling for the interview cohort may introduce selection bias, particularly by underrepresenting patients who did not return for follow-up or those with more acute conditions. Second, the cross-sectional design limits the ability to assess changes in quality of life over time and precludes causal inference. Therefore, the observed association between longer stoma duration and higher quality-of-life scores should be interpreted cautiously as an association rather than evidence of definite longitudinal improvement or adaptation over time. Third, the study was conducted at a single tertiary center, which may affect the generalizability of the findings to other healthcare settings. In addition, certain subgroups, such as patients with temporary loop stomas created in emergencies, may not have been fully captured. The statistical analysis was primarily based on univariable comparisons without multivariable adjustment for potential confounding factors, and although the Vietnamese version of the CoH-QoL-OQ questionnaire underwent translation and pilot testing, formal psychometric validation was not fully performed prior to its application in this study. Despite these limitations, the relatively large overall sample size and the use of a multidimensional disease-specific instrument provide meaningful insights into the real-world experiences of patients living with a stoma and may serve as a foundation for future longitudinal and multicenter studies aimed at improving patient-centered outcomes.

## Conclusions

This study demonstrates that Vietnamese ostomy patients generally reported a relatively favorable quality of life, with higher scores observed in the physical and spiritual domains. In contrast, social well-being remained the most affected dimension, highlighting ongoing challenges in social reintegration. Longer stoma duration was associated with more favorable quality of life scores across several domains, suggesting a possible relationship between longer-term stoma experience and improved adaptation. These findings emphasize the importance of continuous follow-up, patient education, and psychosocial support, particularly during the early postoperative period. Future strategies should focus on improving access to stoma care resources and developing interventions that facilitate social participation and long-term adaptation.

## Figures and Tables

**Figure 1:**
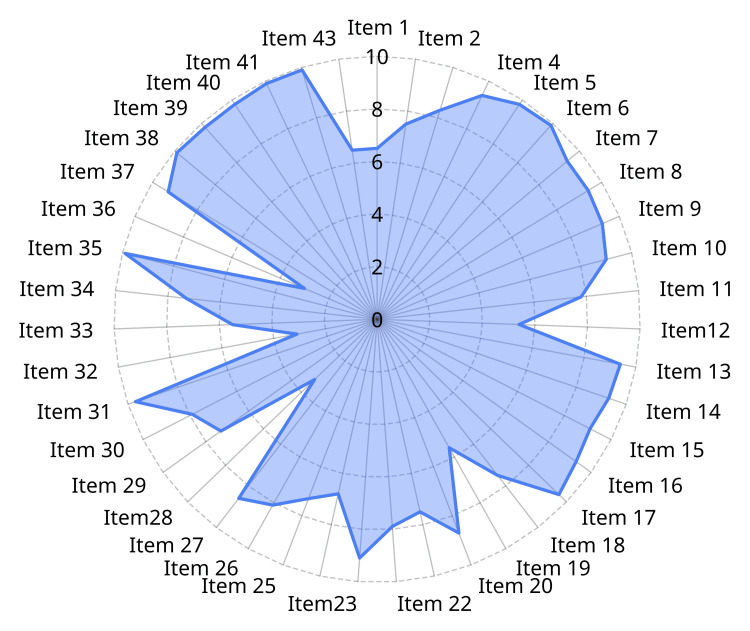
Item-level analysis of quality of life using the City of Hope Quality of Life-Ostomy Questionnaire (COH-QoL-OQ; N=87) The figure was created by the authors using Canva (Canva Pty Ltd., Sydney, Australia).

## Appendices

Appendix A

Original and Vietnamese-Translated Versions of the CoH-QoL-OQ Questionnaire

**Table 5:** Original and Vietnamese-translated versions of the City of Hope Quality of Life-Ostomy Questionnaire (COH-QoL-OQ) The original English version of the CoH-QoL-OQ questionnaire, reproduced from Grant et al. for research purposes. The original authors permit the use and duplication of the questionnaire without additional permission (City of Hope National Medical Center) [11]. The questionnaire was translated into Vietnamese using a standardized forward–backward translation process. The translated version was reviewed by a native English speaker. Any discrepancies were discussed and resolved by the authors, who finalized the Vietnamese version to ensure linguistic accuracy, semantic equivalence, and cultural appropriateness.

The original version of the CoH-QoL-OQ questionnaire	The Vietnamese-translated version of the CoH-QoL-OQ questionnaire which was used in this study
Directions: We are interested in knowing how the experience of having an ostomy affects your quality of life.	Hướng dẫn: Chúng tôi muốn biết trải nghiệm việc mở thông ruột ra da ảnh hưởng đến chất lượng cuộc sống của Ông/Bà như thế nào.
Please answer all of the following questions based on your life at this time.	Vui lòng trả lời tất cả các câu hỏi sau dựa trên cuộc sống của Ông/Bà tại thời điểm này.
Please circle the number form 0 - 10 that best describes your experiences. For example: How difficult is it for you to climb stairs? Not at all difficult 0 1 2 3 4 5 6 7 8 9 10 extremely difficult Circling (2) means you have some but not a lot of difficulty climbing stairs.	Khoanh tròn số từ 0 - 10 mô tả tốt nhất về trải nghiệm của Ông/Bà. Ví dụ: Ông/Bà thấy việc leo cầu thang khó khăn như thế nào? Không khó chút nào 0 1 2 3 4 5 6 7 8 9 10 cực kỳ khó khăn Khoanh số (2) có nghĩa là Ông/Bà gặp một chút nhưng không gặp nhiều khó khăn khi leo cầu thang.
Related to your ostomy, to what extent are the following a problem for you?	Liên quan đến lỗ mở thông ruột ra da, những vấn đề sau đây gây khó khăn cho Ông/Bà ở mức độ nào?
1. Physical strength no problem 0 1 2 3 4 5 6 7 8 9 10 severe problem	1. Sức mạnh thể chất không có vấn đề gì 0 1 2 3 4 5 6 7 8 9 10 có vấn đề nghiêm trọng
2. Fatigue no problem 0 1 2 3 4 5 6 7 8 9 10 severe problem	2. Mệt mỏi không có vấn đề gì 0 1 2 3 4 5 6 7 8 9 10 có vấn đề nghiêm trọng
3. Skin surrounding the ostomy no problem 0 1 2 3 4 5 6 7 8 9 10 severe problem	3. Da xung quanh lỗ mở thông ruột ra da không có vấn đề gì 0 1 2 3 4 5 6 7 8 9 10 có vấn đề nghiêm trọng
4. Sleep disorders no problem 0 1 2 3 4 5 6 7 8 9 10 severe problem	4. Rối loạn giấc ngủ không có vấn đề gì 0 1 2 3 4 5 6 7 8 9 10 có vấn đề nghiêm trọng
5. Aches or pains no problem 0 1 2 3 4 5 6 7 8 9 10 severe problem	5. Đau nhức không có vấn đề gì 0 1 2 3 4 5 6 7 8 9 10 có vấn đề nghiêm trọng
6. Gas no problem 0 1 2 3 4 5 6 7 8 9 10 severe problem	6. Hơi không có vấn đề gì 0 1 2 3 4 5 6 7 8 9 10 có vấn đề nghiêm trọng
7. Odor no problem 0 1 2 3 4 5 6 7 8 9 10 severe problem	7. Mùi hôi không có vấn đề gì 0 1 2 3 4 5 6 7 8 9 10 có vấn đề nghiêm trọng
8. Constipation no problem 0 1 2 3 4 5 6 7 8 9 10 severe problem	8. Táo bón không có vấn đề gì 0 1 2 3 4 5 6 7 8 9 10 có vấn đề nghiêm trọng
9. Diarrhea no problem 0 1 2 3 4 5 6 7 8 9 10 severe problem	9. Tiêu chảy không có vấn đề gì 0 1 2 3 4 5 6 7 8 9 10 có vấn đề nghiêm trọng
10. Leaking from the pouch (or around the appliance) no problem 0 1 2 3 4 5 6 7 8 9 10 severe problem	10. Rò rỉ từ túi (hoặc xung quanh dụng cụ) không có vấn đề gì 0 1 2 3 4 5 6 7 8 9 10 vấn đề nghiêm trọng
11. Overall physical well-being no problem 0 1 2 3 4 5 6 7 8 9 10 severe problem	11. Đối với sức khỏe thể chất tổng thể không có vấn đề gì 0 1 2 3 4 5 6 7 8 9 10 vấn đề nghiêm trọng
12. How difficult has it been for you to adjust to your ostomy? not at all 0 1 2 3 4 5 6 7 8 9 10 a great deal	12. Ông/Bà thấy việc thích nghi với lỗ mở thông ruột ra da của mình khó khăn như thế nào? hoàn toàn không 0 1 2 3 4 5 6 7 8 9 10 rất nhiều
13. How useful do you feel? not at all 0 1 2 3 4 5 6 7 8 9 10 a great deal	13. Ông/Bà cảm thấy mình hữu ích như thế nào? hoàn toàn không 0 1 2 3 4 5 6 7 8 9 10 rất nhiều
14. How much satisfaction or enjoyment in life do you feel? none at all 0 1 2 3 4 5 6 7 8 9 10 a great deal	14. Ông/Bà cảm thấy mức độ hài lòng hay hưởng thụ cuộc sống ở mức nào? hoàn toàn không 0 1 2 3 4 5 6 7 8 9 10 rất nhiều
15. How much are you embarrassed by your ostomy? not at all 0 1 2 3 4 5 6 7 8 9 10 extremely embarrassed	15. Ông/Bà cảm thấy xấu hổ về lỗ mở thông ruột ra da của mình ở mức độ nào? hoàn toàn không 0 1 2 3 4 5 6 7 8 9 10 vô cùng xấu hổ
16. How good is your overall quality of life? extremely poor 0 1 2 3 4 5 6 7 8 9 10 excellent	16. Chất lượng cuộc sống tổng thể của Ông/Bà tốt như thế nào? Rất thấp 0 1 2 3 4 5 6 7 8 9 10 tuyệt vời
17. How is your ability to remember things? extremely poor 0 1 2 3 4 5 6 7 8 9 10 excellent	17. Khả năng ghi nhớ của Ông/Bà thế nào? Rất thấp 0 1 2 3 4 5 6 7 8 9 10 tuyệt vời
18. How difficult is it to look at your ostomy? not at all 0 1 2 3 4 5 6 7 8 9 10 extremely difficult	18. Việc nhìn vào lỗ mở thông ruột ra da của mình khó khăn như thế nào? hoàn toàn không 0 1 2 3 4 5 6 7 8 9 10 rất khó khăn
19. How difficult is it for you to care for your ostomy? not at all 0 1 2 3 4 5 6 7 8 9 10 extremely difficult	19. Ông/Bà thấy việc chăm sóc lỗ mở thông ruột ra da của mình khó khăn như thế nào ? hoàn toàn không 0 1 2 3 4 5 6 7 8 9 10 rất khó khăn
20. Do you feel like you are in control of things in your life? not at all 0 1 2 3 4 5 6 7 8 9 10 completely	20. Ông/Bà có cảm thấy mình đang kiểm soát tốt mọi thứ trong cuộc sống của mình không? hoàn toàn không 0 1 2 3 4 5 6 7 8 9 10 rất tốt
21. How satisfied are you with your appearance? not at all 0 1 2 3 4 5 6 7 8 9 10 extremely satisfied	21. Ông/Bà hài lòng như thế nào về ngoại hình của mình? hoàn toàn không 0 1 2 3 4 5 6 7 8 9 10 vô cùng hài lòng
22. How much anxiety do you have? none at all 0 1 2 3 4 5 6 7 8 9 10 severe	22. Ông/Bà lo lắng đến mức nào? hoàn toàn không 0 1 2 3 4 5 6 7 8 9 10 rất nhiều
23. How much depression do you have? none at all 0 1 2 3 4 5 6 7 8 9 10 severe	23. Ông/Bà bị xuống tinh thần ở mức độ nào? hoàn toàn không 0 1 2 3 4 5 6 7 8 9 10 rất nhiều
24. Are you fearful that your disease will come back? not at all 0 1 2 3 4 5 6 7 8 9 10 extremely fearful	24. Ông/Bà có sợ bệnh của mình sẽ tái phát không? hoàn toàn không 0 1 2 3 4 5 6 7 8 9 10 rất sợ
25. Do you have difficulty meeting new people? not at all 0 1 2 3 4 5 6 7 8 9 10 extremely difficult	25. Ông/Bà có gặp khó khăn khi gặp gỡ những người mới không? hoàn toàn không 0 1 2 3 4 5 6 7 8 9 10 rất khó khăn
26. How much financial burden resulted from your illness or treatment? none at all 0 1 2 3 4 5 6 7 8 9 10 extreme	26. Gánh nặng tài chính do bệnh tật hoặc quá trình điều trị ra sao? hoàn toàn không 0 1 2 3 4 5 6 7 8 9 10 rất nhiều
27. How distressing has your illness been for your family? not at all 0 1 2 3 4 5 6 7 8 9 10 extremely distressing	27. Bệnh tật của Ông/Bà đã làm phiền như thế nào cho gia đình Ông/Bà? hoàn toàn không 0 1 2 3 4 5 6 7 8 9 10 rất nhiều
28. How much does your ostomy interfere with your ability to travel? not at all 0 1 2 3 4 5 6 7 8 9 10 completely	28. Lỗ mở thông ruột ra da của Ông/Bà có ảnh hưởng đến khả năng đi du lịch của Ông/Bà không? hoàn toàn không 0 1 2 3 4 5 6 7 8 9 10 rất nhiều
29. Has your ostomy interfered with your personal relationships? not at all 0 1 2 3 4 5 6 7 8 9 10 completely	29. Lỗ mở thông ruột ra da của Ông/Bà có ảnh hưởng đến các mối quan hệ cá nhân của Ông/Bà không? hoàn toàn không 0 1 2 3 4 5 6 7 8 9 10 rất nhiều
30. How much isolation is caused by your ostomy? none 0 1 2 3 4 5 6 7 8 9 10 a great deal	30. Lỗ mở thông ruột ra da gây ra sự cô lập cho Ông/Bà ở mức độ nào? hoàn toàn không 0 1 2 3 4 5 6 7 8 9 10 rất nhiều
31. Is support from friends and family sufficient to meet your needs? not at all 0 1 2 3 4 5 6 7 8 9 10 extremely	31. Sự hỗ trợ từ bạn bè và gia đình có đủ để đáp ứng nhu cầu của Ông/Bà không? hoàn toàn không 0 1 2 3 4 5 6 7 8 9 10 rất nhiều
32. Has your ostomy interfered with your recreational/sports activities? not at all 0 1 2 3 4 5 6 7 8 9 10 a great deal	32. Lỗ mở thông ruột ra da của Ông/Bà có ảnh hưởng đến các hoạt động giải trí/thể thao của Ông/Bà không? hoàn toàn không 0 1 2 3 4 5 6 7 8 9 10 rất nhiều
33. Has your ostomy interfered with your social activities? not at all 0 1 2 3 4 5 6 7 8 9 10 a great deal	33. Lỗ mở thông ruột ra da của Ông/Bà có ảnh hưởng đến các hoạt động xã hội của Ông/Bà không? hoàn toàn không 0 1 2 3 4 5 6 7 8 9 10 rất nhiều
34. Has your ostomy interfered with your ability to be intimate? not at all 0 1 2 3 4 5 6 7 8 9 10 a great deal	34. Lỗ mở thông ruột ra da có ảnh hưởng đến khả năng thân mật của Ông/Bà không? hoàn toàn không 0 1 2 3 4 5 6 7 8 9 10 rất nhiều
35. Do you have enough privacy at home for doing your ostomy care? not at all 0 1 2 3 4 5 6 7 8 9 10 a great deal	35. Ông/Bà có đủ sự riêng tư ở nhà để chăm sóc lỗ mở thông ruột ra da không? hoàn toàn không 0 1 2 3 4 5 6 7 8 9 10 rất nhiều
36. Do you have enough privacy when traveling for conducting your ostomy care? not at all 0 1 2 3 4 5 6 7 8 9 10 a great deal	36. Ông/Bà có đủ sự riêng tư khi đi du lịch để chăm sóc lỗ mở thông ruột ra da không? hoàn toàn không 0 1 2 3 4 5 6 7 8 9 10 rất nhiều
37. How much uncertainty do you feel about your future? none at all 0 1 2 3 4 5 6 7 8 9 10 extreme	37. Ông/Bà cảm thấy bất an về tương lai của mình không? hoàn toàn không 0 1 2 3 4 5 6 7 8 9 10 rất nhiều
38. Do you sense a reason for being alive? not at all 0 1 2 3 4 5 6 7 8 9 10 a great deal	38. Ông/Bà có cảm thấy cuộc sống có ý nghĩa không? hoàn toàn không 0 1 2 3 4 5 6 7 8 9 10 rất nhiều
39. Do you have a sense of inner peace? not at all 0 1 2 3 4 5 6 7 8 9 10 a great deal	39. Ông/Bà có cảm thấy bình yên nội tâm không? hoàn toàn không 0 1 2 3 4 5 6 7 8 9 10 rất nhiều
40. How hopeful do you feel? not at all 0 1 2 3 4 5 6 7 8 9 10 extremely	40. Ông/Bà cảm thấy hy vọng ra sao? hoàn toàn không 0 1 2 3 4 5 6 7 8 9 10 rất nhiều
41. Is support you receive from personal spiritual activities such as prayer or mediation sufficient to meet your needs? not at all 0 1 2 3 4 5 6 7 8 9 10 completely	41. Sự hỗ trợ Ông/Bà nhận được từ các hoạt động tâm linh cá nhân như cầu nguyện hoặc thiền định có đủ để đáp ứng nhu cầu của Ông/Bà không? hoàn toàn không 0 1 2 3 4 5 6 7 8 9 10 rất nhiều
42. Is support you receive from religious activities such as going to church or synagogue sufficient to meet your needs? not at all 0 1 2 3 4 5 6 7 8 9 10 completely	42. Sự hỗ trợ Ông/Bà nhận được từ các hoạt động tôn giáo như đi nhà thờ hoặc các cơ sở tôn giáo có đủ để đáp ứng nhu cầu của Ông/Bà không? hoàn toàn không 0 1 2 3 4 5 6 7 8 9 10 hoàn toàn đủ
43. Has having an ostomy made positive changes in your life style? not at all 0 1 2 3 4 5 6 7 8 9 10 a great deal	43. Việc mở thông ruột ra da có tạo ra những thay đổi tích cực nào trong lối sống của Ông/Bà không? hoàn toàn không 0 1 2 3 4 5 6 7 8 9 10 rất nhiều

Appendix B

Diagnostic Categories Requiring Stoma Creation

**Table 6:** Diagnostic categories requiring stoma creation

Diagnosis	Interview cohort	Non-interview cohort	Overall cohort
	(N = 87)	(N = 465)	(N = 552)
	n (%)	n (%)	n (%)
Colorectal cancer			
Left-sided colorectal cancer causing bowel obstruction	29 (33.3)	170 (36.6)	199 (36.1)
Right-sided colorectal cancer causing bowel obstruction	1 (1.1)	2 (0.4)	3 (0.5)
Ileostomy created during rectal resection	9 (10.3)	43 (9.2)	52 (9.4)
Abdominoperineal resection (Miles procedure)	8 (9.2)	11 (2.4)	19 (3.4)
Elective Hartmann’s procedure	0 (0)	4 (0.9)	4 (0.7)
Anastomotic leak following colorectal cancer surgery	1 (1.1)	19 (4.1)	20 (3.6)
Anastomotic fistula following colorectal cancer surgery	0 (0)	3 (0.6)	3 (0.5)
Peritonitis due to perforated colorectal cancer	4 (4.6)	20 (4.3)	24 (4.3)
Intra-abdominal abscess due to perforated colorectal cancer	2 (2.3)	6 (1.3)	8 (1.4)
Abdominal wall abscess due to perforated colorectal cancer	0 (0)	4 (0.9)	4 (0.7)
Colonic perforation proximal to colorectal cancer	1 (1.1)	2 (0.4)	3 (0.5)
Cecal perforation due to obstruction from colorectal cancer	0 (0)	6 (1.3)	6 (1.1)
Colorectal cancer with pelvic invasion	0 (0)	6 (1.3)	6 (1.1)
Rectovaginal fistula due to colorectal cancer	0 (0)	6 (1.3)	6 (1.1)
Colovesical fistula due to colorectal cancer	0 (0)	5 (1.1)	5 (0.9)
Small bowel obstruction due to metastasis from colorectal cancer	0 (0)	9 (1.9)	9 (1.6)
Other malignancies			
Rectal fistula due to vaginal cancer	1 (1.1)	0 (0)	1 (0.2)
Colorectal fistula due to bladder cancer	1 (1.1)	1 (0.2)	2 (0.4)
Bowel obstruction due to pancreatic, gastric, or duodenal cancer invading the colon	0 (0)	4 (0.9)	4 (0.7)
Bowel obstruction due to cervical cancer invading the rectum	0 (0)	2 (0.4)	2 (0.4)
Bowel obstruction due to bladder cancer invading the colorectum	0 (0)	1 (0.2)	1 (0.2)
Small bowel obstruction due to metastasis from pancreatic or gastric cancer	0 (0)	3 (0.6)	3 (0.5)
Small bowel obstruction due to metastasis from gynecological malignancies	0 (0)	3 (0.6)	3 (0.5)
Benign bowel diseases			
Left-sided colonic diverticulitis	15 (17.2)	59 (12.7)	74 (13.4)
Right-sided colonic diverticulitis	2 (2.3)	2 (0.4)	4 (0.7)
Small bowel perforation	4 (4.6)	8 (1.7)	12 (2.2)
Colonic perforation	1 (1.1)	11 (2.4)	12 (2.2)
Necrotizing colitis	0 (0)	10 (2.2)	10 (1.8)
Acute mesenteric ischemia (AMI)	1 (1.1)	4 (0.9)	5 (0.9)
Bowel ischemia due to strangulated hernia	0 (0)	3 (0.6)	3 (0.5)
Intestinal obstruction due to tuberculosis	1 (1.1)	2 (0.4)	3 (0.5)
Intestinal perforation due to tuberculosis	0 (0)	3 (0.6)	3 (0.5)
Anastomotic leak following surgery for benign colorectal disease	0 (0)	4 (0.9)	4 (0.7)
Anorectal diseases			
Complex perianal abscess	2 (2.3)	11 (2.4)	13 (2.4)
Complex anal fistula	1 (1.1)	0 (0)	1 (0.2)
Perineal abscess	0 (0)	2 (0.4)	2 (0.4)
Others			
Perineal trauma	0 (0)	5 (1.1)	5 (0.9)
Abdominal trauma	1 (1.1)	1 (0.2)	2 (0.4)
Colorectal foreign body ingestion	0 (0)	5 (1.1)	5 (0.9)
Foreign body insertion into the anus	0 (0)	1 (0.2)	1 (0.2)
Complications related to adnexal abscess surgery	1 (1.1)	2 (0.4)	3 (0.5)
Fecal impaction causing bowel obstruction	1 (1.1)	1 (0.2)	2 (0.4)
Fecal incontinence	0 (0)	1 (0.2)	1 (0.2)

Appendix C

Item-Level Analysis of Quality of Life Using the CoH-QoL-OQ
